# Implementing an Integrated Pharmaceutical Management Information System for Antiretrovirals and Other Medicines: Lessons From Namibia

**DOI:** 10.9745/GHSP-D-18-00157

**Published:** 2018-12-27

**Authors:** David Mabirizi, Bayobuya Phulu, Wuletaw Churfo, Samson Mwinga, Greatjoy Mazibuko, Evans Sagwa, Lazarus Indongo, Tamara Hafner

**Affiliations:** a Pharmaceuticals and Health Technologies Group, Management Sciences for Health, Arlington, VA, USA.; b Systems for Improved Access to Pharmaceutical and Services (SIAPS) Program, Management Sciences for Health, Windhoek, Namibia.; c Division of Pharmaceutical Services, Ministry of Health and Social Services, Windhoek, Namibia.; d SIAPS Program Consultant, Aarhus, Denmark.

## Abstract

The success of the Namibian government's “treatment for all” approach to control and stop the country's HIV epidemic is dependent on an uninterrupted supply of antiretrovirals (ARVs) for people living with HIV. The public health system in Namibia, however, was constrained by an inefficient paper-based pharmaceutical information system resulting in unreliable and inaccessible data, contributing to persistent stock-outs of ARVs and other essential pharmaceuticals. This article describes the incremental implementation of an integrated pharmaceutical management information system to provide timely and reliable commodity and patient data for decision making in Namibia's national antiretroviral therapy (ART) program and the Ministry of Health and Social Services (MoHSS). The system has 4 interlinked information tools: (1) the Electronic Dispensing Tool (EDT) that manages the dispensing and inventory of antiretrovirals at service delivery points; (2) the EDT national database, which facilitates the flow, storage, and collation of ART data at the central level; (3) the Facility Electronic Stock Card used to manage pharmaceutical stocks and report inventory movement data to the national level; and (4) the Pharmaceutical Management Information Dashboard that integrates all 3 tools plus the warehouse management tool used by the central and regional medical stores into 1 dashboard that serves as a platform for the analysis and dissemination of pharmaceutical information throughout the health system. Implementing the pharmaceutical management information system was a prolonged and complicated process, with key challenges related to user acceptance and human resource constraints. The integrated pharmaceutical management information system enables Namibia to collect more than 90% of transactional commodity and patient dispensing data from more than 85% of all ART sites. Health managers use information from the system for medicine quantification decisions and to improve pharmaceutical service delivery. The MoHSS and its partners in the national ART program use the information for monitoring the World Health Organization early warning indicators for HIV drug resistance; ART defaulter tracing; and for planning, reporting, and research purposes. Namibia's pharmaceutical management information system demonstrates the feasibility and benefits of integrating related tools while maintaining their specialized functionality to address country-specific information and inventory management needs.

## Abstract (precis)

Integrating patient and commodity data into one system while maintaining specialized functionality has allowed managers to monitor and mitigate stock-out risks more effectively, as well as provide earlier warning for HIV drug resistance.

## INTRODUCTION

Namibia, an upper middle-income country, is one of the world's most sparsely populated countries. Access to health services is partially constrained by the country's vast geography and the distribution of its population—two-thirds of the population live in sparsely settled rural areas and it is estimated that rural households are 114 minutes away from the nearest public health facility.^1^ A shortage of skilled health personnel, including pharmacy staff, coupled with a fragmented health information system in some areas,^2^^,^^3^ made it difficult for the Ministry of Health and Social Services (MoHSS) to effectively manage decentralized health services across the country's 14 regions.

HIV/AIDS is a leading cause of morbidity and mortality in Namibia. The number of people aged 15 years and older living with HIV is estimated at 260,000, or 12.3% of the total population.^4^ The Namibian government has adopted numerous policies and guidelines to control the HIV epidemic, such as the World Health Organization (WHO) “Treatment for All” approach, which has resulted in high testing and treatment coverage leading to the near elimination of mother-to-child transmission of HIV.^5^ The government has also decentralized antiretroviral therapy (ART) services beyond district hospital and health center pharmacies to include outreach sites, community-based dispensing and prescription refill, and nurse-initiated and nurse-managed ART sites.^6^ The key objective of the 2010–2016 national strategic framework for HIV and AIDS was to achieve universal access to care and treatment for people living with HIV.^7^ These objectives and strategies are ultimately aimed at achieving the broader global goal of 90-90-90, requiring 90% of HIV patients knowing their HIV status, 90% accessing ART, and 90% achieving viral suppression.^8^ To achieve these objectives, Namibia's public health system faces the challenge of sustainably ensuring uninterrupted access to antiretrovirals (ARVs) for 90% or more people living with HIV.

The uninterrupted supply of ARVs requires timely availability of accurate patient and commodity information for decision making. The decentralization of health services brings new challenges in retrieving patient and medicine information from service delivery points. A 2006 assessment found that the lack of computerized information systems was a severe constraint on the management of pharmaceuticals and the tracking of ART defaulters in Namibia.^9^ An earlier assessment found that the paper-based system used at service delivery points—including tertiary, regional, and district hospitals and health centers—was inefficient, contributing to prolonged patient waiting times and a high dispensing workload.^10^ Patient and stock data collected were unreliable, late, and inaccessible for decision making, and some hospitals and health center pharmacies did not routinely collect commodity consumption and stock data.^10^ The MoHSS tried unsuccessfully to improve pharmaceutical inventory management by mandating the use of stock cards in health facilities (MoHSS Circulars 25 and 61). At the regional level, pharmacists routinely lacked access to accurate information and had challenges using the limited information available. In addition, no mechanism for collating and analyzing ART data existed at the central level.

The lack of a system to manage pharmaceutical information meant that ARV quantifications were based on predefined inventory level maximums and minimums, which did not account for current consumption trends or other relevant information.^10^ This led to persistent ARV stock-outs and created an urgent need for an information system to ensure a consistent supply of ARVs to its growing population of people living with HIV.^11^ The findings and recommendations from the assessments^9^^,^^10^ informed the initiation of a now decade-long technical assistance program in Namibia to strengthen pharmaceutical information management for the national ART program and pharmaceutical service delivery.

This article describes the incremental implementation and integration of a pharmaceutical management information system in Namibia to ensure uninterrupted access to ARVs for people living with HIV. The pharmaceutical management information system incorporates components of logistics management information systems (LMIS), but it also includes dispensing, patient, and treatment data, and selected pharmaceutical system performance indicators. The incorporation of these varied sources of data facilitates evidence-based decision making regarding inventory management, resource allocation, pharmaceutical policy, and other aspects of the performance of pharmaceutical service delivery.^12^ An effective pharmaceutical management information system can enhance the availability of and access to information for decision making in Namibia's national ART program and in the Division Pharmaceutical Services (DivPhS) of the MoHSS.

The integrated pharmaceutical management information system incorporates components of logistics management information systems but also includes dispensing, patient, and treatment data to facilitate decision making.

## INTERVENTIONS

**FIGURE 1:**
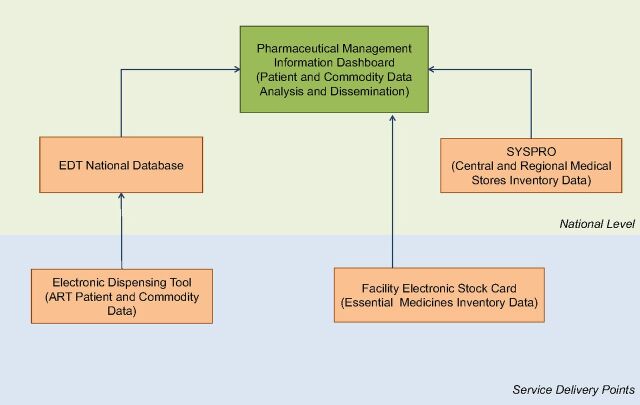
Components of Namibia's Integrated Pharmaceutical Information System Abbreviations: ART, antiretroviral therapy; EDT, Electronic Dispensing Tool.

Between 2007 and 2017, the Strengthening Pharmaceutical Systems (SPS) and Systems for Improved Access to Pharmaceutical and Services (SIAPS) programs, funded by the United States Agency for International Development (USAID), supported the MoHSS with the development and implementation of 4 interlinked pharmaceutical information tools: (1) the Electronic Dispensing Tool (EDT) manages the dispensing and inventory of ARVs at service delivery points; (2) the EDT national database facilitates the flow, storage, and collation of ART data at the central level; (3) the Facility Electronic Stock Card (FESC) is used to manage pharmaceutical stocks and report inventory movement data to the national level; and (4) the Pharmaceutical Management Information Dashboard—a web-based inventory management tool—integrates all 3 tools plus SYSPRO, an inventory management tool used by central and regional medical stores. The dashboard serves as a platform for the analysis and dissemination of pharmaceutical information throughout the health system (Figure 1).

The intervention approach was incremental and evolved to meet the emerging needs of the pharmaceutical system and the national ART program. The intervention started in 2007 with the implementation of the EDT at service delivery points. In 2010, the EDT national database was installed at the central level at the MoHSS. The intervention continued with the implementation of FESC at service delivery points. Finally in 2016, the Pharmaceutical Management Information Dashboard was implemented. Box 1 provides a summary of the system components.

BOX 1 **Description of Components of the Pharmaceutical Management Information System**

- **Electronic Dispensing Tool (EDT).** The EDT is a health facility desktop software for antiretroviral therapy (ART) pharmacy management and reporting. The tool captures ART patient demographic data, dispensing history, and antiretroviral inventory data including stock orders, receipts, issues, and stock taking. It registers daily transactions and produces monthly ART reports that feed into the Pharmaceutical Information Dashboard.
- **EDT National Database.** The EDT National Database is the central repository that aggregates all facility-level individual EDT data sets.
- **Facility Electronic Stock Card (FESC).** The FESC is a simple electronic stock control card. It records data on stock taking, ordering, receiving, issuing, and adjustment of each stocked pharmaceutical item and generates monthly summary stock status reports that feed into the Pharmaceutical Information Dashboard.
- **SYSPRO.** SYSPRO is a proprietary commercial Enterprise Resource Planning software used to manage inventory, supplier and health facility order processing at the central and regional medical stores.
- **Pharmaceutical Management Information Dashboard.** The Pharmaceutical Management Information Dashboard is a web application for collating and visualizing aggregated ART, other essential medicines and commodities, and information on pharmaceutical system performance indicators from various sources including the EDT and the FESC.

An estimated US$4 million was invested over the 10-year period in developing and implementing the pharmaceutical management information system in Namibia. The estimated cost includes software development (20%); capacity building—mainly training, mentoring, and supportive supervision of MoHSS staff at facility, regional, and national levels (50%); equipment (25%); and costs related to data transfer (5%). The SIAPS program covered the initial set-up costs and the Namibian government provided support for human resources and infrastructure. The MoHSS, the Global Fund to Fight AIDS, Tuberculosis and Malaria, and the U.S. Centers for Disease Control and Prevention (CDC) contributed to the purchase of equipment for setting up the system.

### Collection and Collation of Patient and Commodity Data

#### Electronic Dispensing Tool

The EDT was implemented in 2007 at service delivery points to manage the dispensing and inventory of ARVs. ARV prescription filling is the primary source data for EDT, which captures ART uptake patterns (patients that are new, active, and lost to follow-up), patients' pill counts and appointment keeping, medicines coverage, and availability of ARVs at the health facility. These data enable better monitoring of patients' adherence to medications and WHO early warning indicators for HIV drug resistance. The intervention started with the identification of “quick adopters”—pharmacists and pharmacy assistants who were technologically interested in using computers and electronic tools. The EDT was first piloted at 10 district-level and referral hospitals over a 3-month period in 2007, and then rolled out to 20 hospitals and large health centers in the next 6 months. Within 1 year, all 34 hospitals and 2 high-volume health centers were using the EDT.

Within a year, all 34 hospitals and 2 high-volume health centers were using the Electronic Dispensing Tool to manage the dispensing and inventory of ARVs at service delivery points.

In Namibia, 6 of 10 people live in rural communities with limited access to ART services.^13^ The government therefore adopted a decentralized ART model—nurse-initiated and nurse-managed services at primary health care clinics—to bring ART services closer to these rural communities. The paper-based reporting systems used at these sites had discrepancies in the number of patients accessing treatment and most of the patients were falsely tagged as lost to follow-up in the EDT national database. These inaccurate data negatively affected ART program planning at the national level.

To address data and patient management challenges in rural areas, SIAPS adapted EDT to a portable handheld device referred to as a mobile EDT (mEDT).^11^ In 2013, pharmacy staff and other health workers were trained on capturing ARV dispensing and stock data during outreach activities in communities using the mEDT. Mobile EDT devices were also deployed for use at smaller primary health care clinics where computerization was not feasible. After 2 years of implementation, a patient referral module was implemented that enabled district hospitals to refer patients downwards to outreach sites and smaller clinics to pick up refills using mEDT. A total of 61 mEDTs have since been deployed and 70 health workers at 51 primary health care sites in select regions with high HIV prevalence have been trained to use the mEDT. Each mEDT is affiliated with a designated main (parent) EDT site. The data collected through mEDT are periodically synchronized with the EDT at the main site. Pharmacy staff were initially trained and later retrained on the collection, collation, and interpretation of the EDT data for their health facilities and districts.

The program adapted the Electronic Dispensing Tool to a portable handheld device to reach communities in rural areas.

#### Electronic Dispensing Tool National Database

The EDT national database was implemented in 2010 to aggregate and summarize EDT data at the central level.^11^ Encrypted data from EDT at service delivery points are automatically transmitted through low-cost mobile telecommunication 3G devices directly to the national database. This reduces the expense of the physical transfer of data from the facilities to the central level and eases the burden of producing paper reports. The national database facilitates a national backup of data from service delivery points and the quick generation of up-to-date data sets for analysis and evidence-based decision making.

#### The Facility Electronic Stock Card

The intervention continued with the implementation of the FESC at health facilities. Based on the experience with the EDT, the MoHSS expressed the need for an electronic tool to help improve the management and availability of other essential commodities in the health system. In response, SIAPS supported the development of the FESC and its implementation in 51 service delivery points—pharmacies at 35 hospitals, 6 health centers, and 10 clinics. Following the implementation of the FESC in 2016, 138 pharmacy staff were trained on how to use the FESC for managing inventory and generating and interpreting LMIS reports. The FESC captures pharmaceutical stock and inventory transaction data, automatically computes average monthly consumption and minimum and maximum stock quantities, and provides suggested ordering quantities. The FESC also includes a module for making positive and negative adjustments and explaining the reason for such adjustments. It generates LMIS reports summarizing stock status, issue by item, issue by facility, receiving, and Central Medical Stores (CMS) service levels to a specific facility.

### Analysis and Dissemination of Patient Dispensing and Commodity Data

#### Pharmaceutical Management Information Dashboard

In 2016, the Pharmaceutical Management Information Dashboard was implemented to serve as a platform for the analysis and dissemination of information from the other tools and to improve access to information for decision making. Although the EDT and national database existed, information was still not easily accessible for decision making. Pharmaceutical and program managers relied on quarterly reports manually generated by DivPhS for information about patients and commodities in the national ART program. Information access was further constrained by having to submit requests to DivPhS for specific information from the national database. The MoHSS had implemented SYSPRO, a proprietary commercial Enterprise Resource Planning software, at the CMS in 2001, and later at the 2 regional medical stores. The central and regional medical stores use SYSPRO to manage inventory, supplier procurement order, and health facility order processes, but program managers and facility-level managers had no readily available information about stock levels of essential medicines at the medical stores. Concurrent with the implementation of FESC, SIAPS supported the implementation of the Pharmaceutical Management Information Dashboard, which consolidates data from SYSPRO, the EDT, FESC, and EDT national database to automatically compute selected indicators and generate standardized reports summarizing commodity and patient information (Figure 1).

The dashboard has 3 modules: ART, essential medicines and clinical supplies, and service performance. The ART module provides summary reports including the number and trend of active patients and new patients accessing HIV treatment, retention and adherence rates, and early warning indicators of HIV drug resistance (Box 2). The service performance module provides information on 22 essential pharmaceutical service performance indicators that describe the status of pharmaceutical service delivery nationwide. Reports from FESC feed into the essential commodities module, which provides an overview of stock status for essential medicines and clinical supplies at district-level facilities across the country.

BOX 2 **Summary of Reports Available on the ART Module of the Pharmaceutical Management Information Dashboard**

**Commodity-Related:**

- ARV stock status at national, regional, district, and facility levels
- National ARV stock pipeline
- National ARV stock status by risk category
- ARV stock status trend by risk category of facilities with a stock-out of HIV tracer commodities
- Value of ARV expiries and damages
- Percentage of facilities with a stock-out of key items

**Treatment-Related:**

- Trend in backbone ARV used to initiate ART
- Regimens used to initiate ART
- ARVs consumption trends
- Consumption trends for the top 5 ARVs

**Patient-Related:**

- Percentage of patients at risk of medicine shortages and stock-outs
- Trend of patients on ART
- ART patient uptake by region
- ART patients by regimen
- Comparative trend of patients on ART
- ART patients by regimen category
- Patients by duration on treatment
- Patients distribution by age group
- Monthly patient status

**Retention and Adherence:**

- Adult and pediatric patient adherence to ART by pill count
- National (aggregate) adherence to ART by pill count
- Trend in retention rates
- ART Retention and loss to follow-up rates
- Retention rates on ART by region
- Retention rates on ART for patients by facility
- Number of pickups per day
- Regimen switch

**FIGURE 2:**
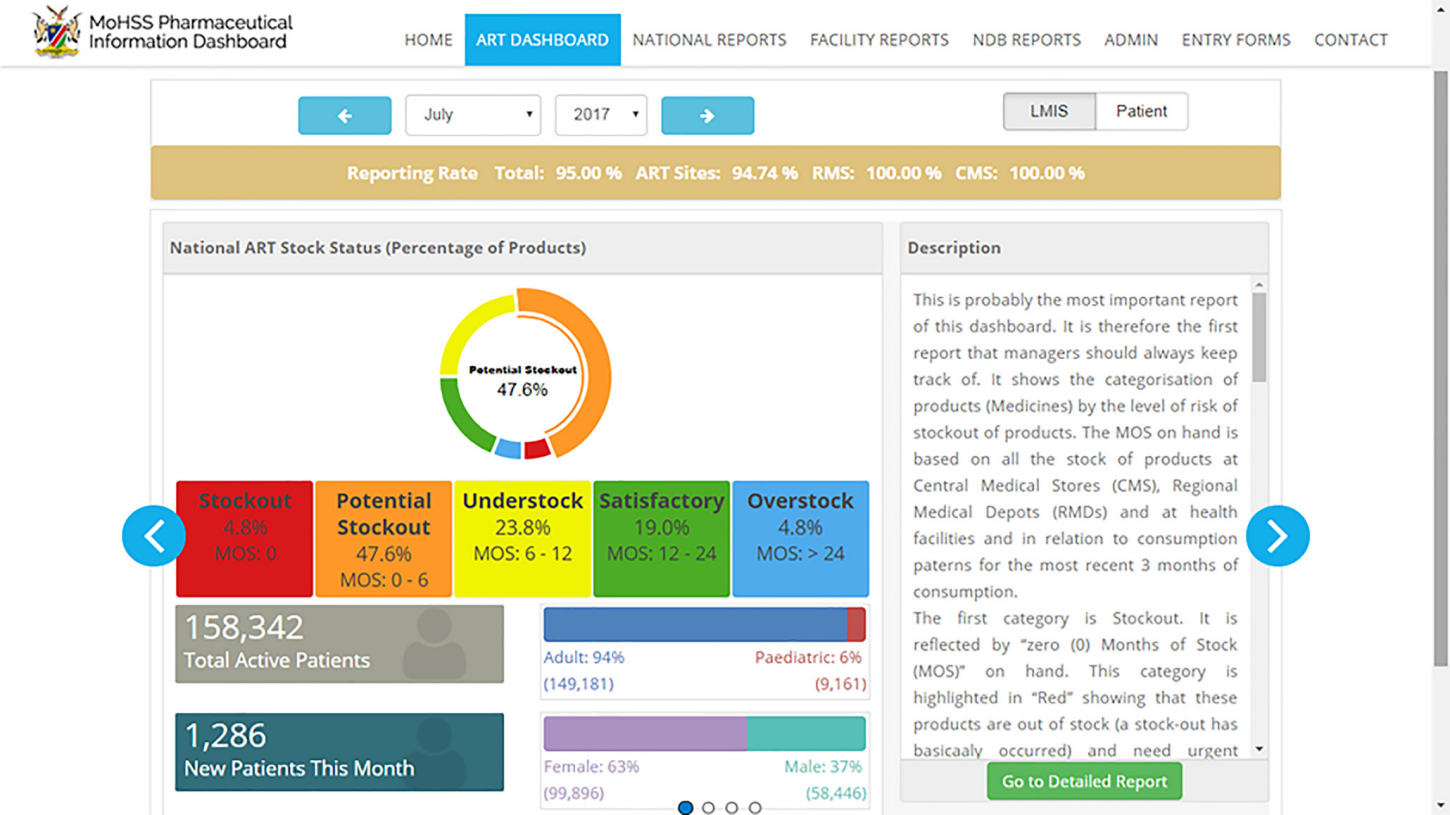
Screenshot of ART Module of the Pharmaceutical Management Information Dashboard Reporting the National ART Stock Status, July 2017 Abbreviation: ART, antiretroviral therapy.

A key feature of the dashboard is an incorporated early warning system to alert supply chain managers of potential stock-outs of medicines, vaccines, and clinical supplies. This makes it easier for managers to identify potential stock-outs or overstocking, and it triggers commodity redistribution to avoid stock-outs or expiries. More than 50 standardized reports are available on the dashboard. One of the most important reports is the national ART stock status, which is disaggregated by risk levels (stock-out, potential stock-out, understock, satisfactory, and overstock). As shown in Figure 2, this risk profile allows easy visualization of stock status nationally for a defined time period. ART stock-out rates can also be displayed geographically for a given month or as a time series. Depending on the indicator or report, users can customize data aggregation by period, facility, region, commodity group, or patient group. For example, trends in new and active ART patients can be disaggregated into adult and pediatric patients and by gender.

The Pharmaceutical Management Information Dashboard consolidates 4 information tools and provides an early warning system to alert managers of potential stock-outs.

Training technical and program managers on the use of the dashboard was a critical component of the dashboard implementation in 2016. At the national level, managers from DivPhS, Directorate of Special Programs, and partner organizations were oriented on the type of data available and accessing and using these data for decision making. At the regional level, SIAPS conducted quarterly workshops on EDT, FESC, and the dashboard to enable regional management teams to understand the purpose of the tools, strengthen their competency in supervising the use of the tools, and using the information generated by the tools for making decisions at the regional and district levels. Clinical mentors in the national ART program were also trained to strengthen their capacity to use information from the dashboard for clinical and programmatic decision making. The ART Logistics Pharmacist in DivPhS, with technical support from SIAPS, served as the dashboard administrator, approving user access and using activity logs to monitor dashboard access and use.

## METHODS

Our article relied on a desk review of documents produced by SIAPS and its predecessor programs, including assessment and technical reports relevant to Namibia's national ART program. In addition, we reviewed data and reports from the Pharmaceutical Management Information Dashboard to summarize average reporting rates and trends. A literature search was conducted with Google Scholar and PubMed for peer-reviewed publications on HIV treatment and care that included analysis of data from the EDT. Keywords in various combinations included Namibia, antiretroviral (*ARV, ART), HIV, drug resistance, HIVDR, and electronic dispensing tool (*EDT). Information from these sources was used to develop a narrative of the implementation process, the challenges, and some of the lessons learned. SIAPS technical staff reviewed drafts of the narrative and provided input as needed for correction and clarification.

## RESULTS

With the dashboard, Namibia has successfully achieved integration of all its pharmaceutical information tools (Figure 1). This integrated pharmaceutical management information system enables Namibia to collect more than 90% of commodity and patient dispensing data from more than 85% of all ART sites within 15 days of each month. With proper and consistent reporting and use, technical service and program managers can access patient and commodity data with only a 1-month lag. Below are some examples of how information from the pharmaceutical management information system is used at service delivery points and at regional and national levels.

### Service Delivery Points and Regional Levels

Pharmacy staff in 64 ART facilities currently use the EDT to manage more than 160,000 patients. Pharmacy staff, including nurses and community health workers, use mEDT to track 12,293 patients, which is approximately 10% of ART patients in Namibia who are managed at nurse-initiated and nurse-managed sites. Between April 2017 and March 2018, the average ART module reporting rate to the dashboard was 97% (Table), with 10% of facilities submitting late reports.

**TABLE.** Facility Reporting Rates on the Dashboard, April 2017 to March 2018

Month	ART (%)	FESC (%)
April 2017	100	62
May 2017	89	49
June 2017	98	54
July 2017	100	62
August 2017	95	65
September 2017	100	73
October 2017	95	62
November 2017	95	62
December 2017	100	73
January 2018	98	62
February 2018	98	57
March 2018	97	55
**Average**	97	**61**

Abbreviations: ART, antiretroviral therapy; FESC, Facility Electronic Stock Card.

The average rate of facilities reporting ART module information to the dashboard was 97%.

FESC, which was more recently introduced, is still gaining traction. Some facilities have demonstrated efficiency gains with consistent use of the tool.^14^ However, other facilities have failed to use the tool consistently to track stock levels, or they are submitting reports late or inconsistently. The average FESC reporting rate for facilities to the dashboard between April 2017 and March 2018 was 61% (Table), with 50% of facilities reporting late. Reporting rates and usage are expected to increase as users become more familiar with FESC. Regardless, there has been an improvement in inventory control and quantification—the percentage of facilities with stock within minimum and maximum levels increased from 16% in 2011 to 67% in 2018.^15^^,^^16^ The pharmaceutical information tools, along with other factors, contributed to this improvement.

Regional pharmacists are expected to use the EDT and FESC for dispensing and inventory management in their capacity as pharmacists for the regional hospitals. Regional pharmacists also serve an administrative function by ensuring support to service delivery points, managing the flow of data to the national level, and ensuring the use of the tools at service delivery points. Functions related to data management at the regional level have not been clearly defined, which has constrained information use for decision making at this level.

### National Level

DivPhS managers use the information for improving service delivery and making quantification and procurement decisions. Consumption and stock level data from the dashboard were critical inputs for the latest ARV quantification done for the period October 2016 to March 2019. The dashboard has filled a critical information gap for DivPhS managers at the national and regional levels for monitoring stock levels. Before the dashboard, the CMS had no access to comprehensive information about stock levels at regional medical stores and at service delivery points. The CMS had to call individual facilities for stock counts when it needed to know stock levels across the country and few facilities reported any stock on hand when ordering new stock. There was no structured way of compiling stock status at the facility level for meaningful discussions and designing strategies at the national level. That information is now readily available on the dashboard.

In cases where there is an impending stock-out or change in treatment guidelines, managers can use the dashboard to identify regions or facilities with stock levels above the maximum buffer stock needed and redistribute accordingly to avert a stock-out or avoid wastage. Pharmacists across the country have formed an interactive group on the WhatsApp social media platform where they discuss their analysis of reports from the dashboard, enabling them to easily locate and redistribute stock. However, managers are still adapting to the nascent dashboard and learning to make full use of its capabilities to incorporate the data in daily practices and decision making. Further, the CMS has been beleaguered by recent human resources shortages, which have limited their use of the information and dashboard in general.^17^

Within the national ART program, the MoHSS and its partners have been using information from the tools for ART defaulter tracing, planning, reporting, and operational research. The EDT is the most reliable source of information on ART adherence in Namibia. The data are used to identify ART patients lost to follow-up for patient tracing in the Namibia Adherence and Retention Program.^18^ Namibia uses the EDT system to monitor timely pill pick-up, retention in care, pharmacy stock-outs, and dispensing practices—all HIV drug resistance early warning indicators. The annual monitoring of early warning indicators has led to public health recommendations and action on increasing defaulter tracing, improving ART record systems, and scaling up differentiated models of care.^19^ MoHSS clinical managers also use data to inform decisions regarding adherence, treatment guidelines, and decentralization of ART programs. They use the distribution of ART regimens to monitor the number of patients maintained on failed treatments and to identify potential noncompliance with standard treatment guidelines. They also use the new and total ART patient data to identify high congestion sites and inform decisions in the clinical mentor program and the decentralization of the national ART program.

Namibia uses the Electronic Dispensing Tool to monitor HIV drug resistance early warning indicators.

The Research, Monitoring and Evaluation Unit in the Directorate of Special Programs and the Joint United Nations Programme on HIV/AIDS (UNAIDS) use the EDT data to formulate estimates on HIV prevalence and incidence in Namibia. These estimates are used for various internal and external reporting and planning purposes.^4^^,^^20^ USAID and the CDC use the data, including the ARV refill and number of newly initiated ART patients data, to estimate ART retention when developing country operational plans for the U.S. President's Emergency Plan for AIDS Relief.^20^^,^^21^ UNAIDS also uses the data from EDT to track progress on United Nations Assembly commitments to HIV and to help identify focus population groups for expanding treatment and adherence at the regional and global levels. The use of EDT data has contributed to Namibia being identified as a country in the Eastern and Southern Africa region on target to reducing the number of people newly infected with HIV and dying from AIDS-related causes.^22^

Fourteen peer-reviewed articles using EDT data have been published and some are linked to decision making in the national ART program (Box 3). An assessment of HIV drug resistance early warning indicators has led to changes in recordkeeping and the strengthening of adherence and monitoring.^24^ The MoHSS and researchers have used EDT data to investigate adverse reactions associated with zidovudine, tenofovir, and nevirapine.^23^^–^^25^ The findings from the nevirapine study contributed to the MoHSS revising its treatment guidelines and stopping the use of nevirapine-containing ART to initiate treatment of pregnant women with high baseline CD4 cell counts.^25^

BOX 3 **Peer-Reviewed Publications Using Electronic Dispensing Tool Data**

- Hong SY, Jonas A, Dumeni E, et al. Population-based monitoring of HIV drug resistance in Namibia with early warning indicators. *J Acquir Immune Defic Syndr.* 2010;55(4):27-31. CrossRef. Medline
- Corbell C, Katjitae I, Mengistu A, et al. Records linkage of electronic databases for the assessment of adverse effects of antiretroviral therapy in sub‐Saharan Africa. *Pharmacoepidemiol Drug Saf*. 2012;21(4):407–414. CrossRef. Medline
- Hong SY, Jerger L, Jonas A, et al. Medication possession ratio associated with short-term virologic response in individuals initiating antiretroviral therapy in Namibia. *PLos One.* 2013;8(2):e56307. CrossRef. Medline
- Jonas A, Gweshe J, Siboleka M, et al. HIV drug resistance early warning indicators in Namibia for public health action. *PLoS One*. 2013;8(6):e65653. CrossRef. Medline
- McQuide PA, Kolehmainen-Aitken RL, Forster N. Applying the workload indicators of staffing need (WISN) method in Namibia: challenges and implications for human resources for health policy. *Hum Resour Health*. 2013;11(1):64. CrossRef. Medline
- Sagwa E, Ruswa N, Musasa JP, Mantel-Teeuwisse AK. Adverse events during treatment of drug-resistant tuberculosis: a comparison between patients with or without human immunodeficiency virus co-infection. *Drug Saf*. 2013;36(11):1087–1096. CrossRef. Medline
- Hong SY, Fanelli TJ, Jonas A, et al. Household food insecurity associated with antiretroviral therapy adherence among HIV-infected patients in Windhoek, Namibia. *J Acquir Immune Defic Syndr*. 2014;67(4):e115-e122. CrossRef. Medline
- Jonas A, Sumbi V, Mwinga S, et al. HIV drug resistance early warning indicators in Namibia with updated World Health Organization guidance. *PLoS One*. 2014;9(7):e100539. CrossRef. Medline
- Hong SY, Jonas A, DeKlerk M, et al. Population-based surveillance of HIV drug resistance emerging on treatment and associated factors at sentinel antiretroviral therapy sites in Namibia. *J Acquir Immune Defic Syndr.* 2015;68(4):463-471. CrossRef. Medline
- Kalemeera F, Mengistu AT, Gaeseb J. Tenofovir substitution in Namibia based on an analysis of the antiretroviral dispensing database. *J Pharm Policy Pract*. 2015;8(1):14. CrossRef. Medline
- Asefa W, Tsegaye D, Dalebout S, Ahmed E. Improving adherence to antiretroviral therapy in Namibia. *Health Aff (Millwood)*. 2016;35(12):2348. CrossRef. Medline
- Kalemeera F, Mbango C, Mubita M, Naikaku E, Gaida R, Godman B. Effect of changing from first- to second-line antiretroviral therapy on renal function: a retrospective study based on data from a single health facility in Namibia. *Expert Rev Anti Infect Ther*. 2016;14(8):777–783. CrossRef. Medline
- Kalemeera F, Mengistu AT, Gaeseb J. Assessment of the nevirapine safety signal using data from the national antiretroviral dispensing database: a retrospective study. *J Pharm Policy Pract*. 2016;9:5. CrossRef. Medline
- Mutenda N, Bukowski A, Nitschke AM, et al. Assessment of the World Health Organization's HIV drug resistance early warning indicators in main and decentralized outreach antiretroviral therapy sites in Namibia. *PLoS One*. 2016;11(12):e0166649. CrossRef. Medline

## CHALLENGES AND LESSONS LEARNED

Migrating from a paper-based pharmaceutical management information system to an electronic system was a prolonged and complicated process with challenges related to behavioral, technical, and organizational factors. These challenges are highlighted in the sections that follow, along with lessons learned.

### Gaining User Acceptance of the Tools

Negative attitudes toward technology and resistance to change were perhaps the biggest challenges. In the beginning, there was no culture of using computerized information technology to provide care in the health system and some health workers were not in favor of abandoning the paper-based system. Further, health workers were not accustomed to using data for decision making. As such, health workers and managers did not consider use of the electronic system as a priority or as an advantage. The paper-based system was the official system for audit and accountability purposes and provided no incentive for adopting the electronic system.

From the start of the implementation, SIAPS identified pharmacists and pharmacy assistants who were interested in using computers and electronic tools and empowered them as early adopters and ambassadors of the tools. The tools were developed with substantial input from pharmacy staff, which created ownership and interest in the success of the tools. Further, tool development took into account existing processes and procedures of collecting and reporting pharmaceutical information. The progressive implementation of the tools facilitated substantial stakeholder involvement and gradual user adoption without staff being overwhelmed.

Political will and ownership from the government were critical enabling factors in gaining user acceptance of the integrated pharmaceutical management information system. The system evolved over time to address specific needs identified by the MoHSS, which generated immense political support and ownership of the tools by the ministry. The EDT became an integral part of ART dispensing at service delivery points, and an indispensable source of timely and reliable data for the national ART program and pharmaceutical managers. The early success of the EDT created an environment conducive to engage stakeholders at all levels for the implementation of FESC and the dashboard. Engagement with the MoHSS minister and permanent secretary, in particular, and their leadership and political commitment were pivotal in the successful adoption and implementation of the tools. The tools gained legitimacy when the MoHSS leadership adopted them as the official systems for audit and accountability purposes, with DivPhS owning and managing the tools. Continuous engagement with the MoHSS; alignment of the tools to government priorities, process, and systems; and a persistent effort to sustain interest and leadership were critical for building political will and government ownership, which in turn encouraged acceptance of the tools.

Political will and ownership from the government were critical enabling factors in gaining user acceptance of the integrated pharmaceutical management information system.

### Strengthening Human Resource Capacity

Namibia has depended heavily on foreign pharmacy staff because of a national shortage of trained health workers. As a result, a high pharmacy staff turnover and low technical capacity of available staff resulted in lack of human resources being a key challenge. SIAPS adopted a multipronged approach to address this human resource challenge. To strengthen the technical capacity of the pharmacy staff, SIAPS worked with the MoHSS to conduct regular trainings on the tools, complemented with continuous mentorship through supportive supervisory visits to pharmacy staff at all health facilities.^15^^,^^16^ To build institutional memory and overcome challenges related to high staff turnover, SIAPS added a video training module to the EDT to provide new users with a step-by-step orientation. Further, all the tools have a simple user-friendly interface, which facilitates tool adoption among all cadres of health workers. The simplicity of the EDT, for example, means that mentorship from colleagues is sufficient to help new pharmacy staff quickly learn and use the tool for dispensing without formal training.

To address the bigger challenge of pharmacy staff shortages, SIAPS collaborated with local training institutions for pharmacists and pharmacist assistants, incorporating the tools into the curricula and extending training to the different cadres of pharmacy staff. This has increased the pool of potential recruits who are familiar with the tools and have the technical capacity to use them effectively for patient management. It is clear that training users has to be an ongoing process and should be incorporated in the preservice curriculum to ensure sustainability of the tools.

### Financial and Technical Sustainability

Substantial costs were associated with the procurement, installation, and maintenance of the equipment and software required for the integrated pharmaceutical management information system. Development partners funded the initial procurement, development and installation of the system hardware and software, and the Namibian government agreed to take over the full cost of operating and maintaining the system once SIAPS support ended. Reductions and restrictions on budgetary allocations to the MoHSS, however, meant that the government was unable to fully finance the system. USAID therefore provided temporary support to give the MoHSS more time to fully absorb the costs. The financial sustainability of the system will depend on the MoHSS's success in getting future budget requests approved.

The MoHSS's centralized information technology (IT) department took over installation and maintenance of the system. However, no staff in the MoHSS IT department was assigned specific responsibility for the administration and technological support for pharmaceutical information tools implemented by the project. Further, the DivPhS at the national level, which owns and manages the tools, had no IT personnel to service and maintain the tools. The DivPhS therefore had little capacity to handle the technical management of the tools at the national level while the districts and regions had limited support for health information systems. SIAPS partially addressed this issue by seconding and transitioning IT staff to the MoHSS to provide dedicated support for the tools. SIAPS also collaborated with local academic institutions to train both pharmacy and IT students to strengthen the technical capacity of potential recruits for maintaining the tools.

Limited Internet connectivity in some public-sector pharmacies across the country, software problems, the lack of IT staff at the MoHSS dedicated to the tools, and the lack of a structured replacement plan for broken or obsolete computers and printers in the facilities continue to present technical challenges. At the start of the intervention, poor Internet connectivity in some regions hampered the use of the tools and the electronic transmission of data was a substantial challenge. Internet connectivity was improved through a partnership with a local telecommunications company, which automated data transmission on a simple, low-cost, secure, and efficient wireless area network platform using 3G devices that were widely available across Namibia. The transmission of ART and FESC data is currently dependent on a reliable Internet network. The MoHSS has rolled out new integrated local networks. The sustainability of the pharmaceutical management information system will partially depend on the MoHSS's ability to maintain and update the system's equipment and software, fund Internet service for the facilities, and ensure access to the new local network.

### Interoperability and Integration

In 2012, 61 health information system tools, with varying degrees of functionality and use, were operating within the Namibian health system.^2^ Most of these systems were, and still are, unable to share data. The pharmaceutical management information system therefore serves as an example of how Namibia can work toward integrating its other tools. However, this fragmentation also presents challenges for the sustainability and integration of the pharmaceutical management information system in the broader national health information system. There is also an ongoing e-Governance initiative through which the Namibian government has been using information technologies, including integrated local networks and mobile computing, to expand service delivery to citizens and improve coordination and communication between government departments. The MoHSS's ability to integrate the tools into the e-Governance initiative could help mitigate some of the fragmentation problems.

### Continuous Monitoring and Data Quality

The success and sustainability of the pharmaceutical management information system hinge on the quality of the data and the required human resources and technical capacity to maintain the system and support its use. The ART logistics pharmacist serves as the administrator for the dashboard and follows a data verification and monitoring protocol, which includes sending reminders to facilities to submit facility-level reports. In the early stages of dashboard implementation and adoption, data requested by MoHSS managers and development partners to analyze the various trends associated with treatment interventions and stock status helped to identify gaps and errors in the data. Identification of any errors and gaps further prompted the pharmacist to follow up with the relevant facilities for clarification and requests for additional data, which over time helped to improve the quality of the data on the dashboard. Further, this feedback process between pharmacists, health staff and program managers, and facilities demonstrated the usability of the tool and contributed to facility-level ownership of the tools.

FESC and EDT were developed to capture real-time transactional data with regular automated electronic updates to the national database. Further, the database query has a predefined list of commonly required and critical reports that are easily retrievable and customizable to respond to key information needs and support decision making. Data are now readily available on the dashboard with only a 1-month lag, allowing managers clear visibility of the consumption and stock level trends throughout the country. The real-time data capture and automated updates help to eliminate much of the workload and task shifting associated with compiling and submitting reports. However, the suboptimal use of FESC threatens the quality of data in the pharmaceutical management information system. Going forward, the MoHSS needs to clarify roles and responsibilities for pharmaceutical information use and actively promote the use of information in decision making, including at the regional level. The MoHSS must also allocate the resources necessary to increase the technical capacity and human resources needed to manage and use the integrated system. National-level ownership of the tools cannot be over-emphasized, but without facility-level ownership and use, the tools' sustainability and data quality will be undermined.

A feedback process between pharmacists, health staff and program managers, and facilities demonstrated the usability of the tool and contributed to facility-level ownership.

### System Replication

SIAPS has supported the implementation of similar but less comprehensive systems in other countries. In Bangladesh, the SIAPS program supported the Directorate General of Family Planning to implement an electronic logistics management information system, which enables health program managers to monitor stock levels at the facility level and identify stock-out risks.^26^ Similarly, SIAPS supported the Mali Ministry of Health, Sanitation, and Hygiene to develop and implement a pharmaceutical web-based dashboard called OSPSANTE for capturing, tracking, and aggregating patient and health commodity data.^27^ As in Namibia, the systems in Bangladesh and Mali combine patient and commodity data, making data more accessible and visible for the management of pharmaceutical products. The systems have contributed to the reduction of stock-outs and improved medicines availability. These examples demonstrate that the approach used in Namibia is replicable in low-income countries, not only for ART but also other pharmaceutical products and health programs. The partnership with the local telecommunications company in Namibia to automate electronic data transmission using a 3G wireless area network is worth considering in low-resource settings with limited Internet connectivity and good 3G or mobile telecommunications data coverage.

### Limitations

This article has several limitations. It does not include many quantifiable measures to demonstrate a direct effect of the implementation and use of the tools (particularly FESC and the dashboard) on pharmaceutical service delivery and the national ART program outcomes. FESC and the dashboard are still being institutionalized and the pharmaceutical management information system has yet to undergo a full assessment that includes direct measures. As a result, the article focuses on the implementation and integration of the system and the lessons learned from that process and less on outcome measures. However, this article demonstrates how the integrated information system has streamlined the collection, collation, and analysis of pharmaceutical information and the resulting improvements in the availability and accessibility of pharmaceutical information for evidence-based decision making at the different levels of the health system. The lessons learned from the implementation process will help to inform strategies adopted by practitioners and policy makers aiming to improve the management of pharmaceutical information.

## CONCLUSIONS

The management of pharmaceutical information is critical for improving population health outcomes. The collection and use of health and logistics data facilitate accurate quantification and procurement of medicines and financial planning, thus ensuring an uninterrupted supply of pharmaceutical products. This in turn helps improve access to medicines and contributes to better health outcomes. Namibia's integrated pharmaceutical management information system seamlessly merges patient and commodity data and creates a common information platform for decision making. The system has improved the availability and visibility of pharmaceutical information in Namibia, contributing to more reliable quantifications and facilitating better inventory management of ARVs and other pharmaceutical products. Further, the system provides a reliable means for managing ART patients and monitoring ART adherence and HIV drug resistance early warning indicators. The incremental implementation of the tools resulted in a simple and practical system that meets the specific and emerging needs of the national ART program and the MoHSS. Ultimately, this type of integrated system makes it easier to identify discrepancies between commodity supply and demand and can strengthen transparency and accountability in the pharmaceutical system. Despite some of the challenges highlighted, the implementation of the integrated pharmaceutical management information system in Namibia demonstrates a feasible approach for integrating separate existing information tools at different levels of the system while maintaining their unique functions.

## Notes

**Peer Reviewed**

**Funding:** This work was supported by the Systems for Improved Access to Pharmaceuticals and Services (SIAPS) Program, which is funded by the U.S. Agency for International Development (USAID), under the terms of cooperative agreement number AID-OAA-A-11-00021.
